# Evolution-driven crosstalk between glioblastoma and the tumor microenvironment

**DOI:** 10.20892/j.issn.2095-3941.2022.0771

**Published:** 2023-03-08

**Authors:** Lingxiang Wu, Ruichao Chai, Zihan Lin, Rongrong Wu, Diru Yao, Tao Jiang, Qianghu Wang

**Affiliations:** 1 Department of Bioinformatics, Nanjing Medical University, Nanjing 211166, China; 2 Institute for Brain Tumors, Jiangsu Collaborative Innovation Center for Cancer Personalized Medicine, Nanjing Medical University, Nanjing 211166, China; 3 Department of Molecular Neuropathology, Beijing Neurosurgical Institute, Capital Medical University, Beijing 100070, China; 4 Chinese Glioma Genome Atlas Network (CGGA), Beijing 100070, China; 5 Department of Neurosurgery, Beijing Tiantan Hospital, Capital Medical University, Beijing 100070, China

Glioblastoma (GBM) is a malignant adult brain tumor for which 90% of patients experience recurrence within a year after surgery^1^. Evolution confers treatment resistance capabilities on tumors^2^. The diversification of malignant and non-malignant (i.e., stromal and immune cell) compartments in the tumor microenvironment (TME) during tumor evolution^3–7^ eventually results in the formation of a complex interaction network that promotes tumor progression.

## Tumor evolution endows GBM with the plasticity to adapt to environmental stresses

Numerous studies^8–11^ have demonstrated that GBM exhibits a variety of molecular states associated with specific genomic variation and TME characteristics. Using single-cell tracing technology, Neftel et al.^11^ have demonstrated that tumor cells in a single state can form a tumor block consisting of multiple cellular states after being implanted in mice, thus indicating that these tumor cells have high plasticity and can dynamically transform cell states. Our recent study^12^ has indicated that the traits formed during natural tumor evolution are associated with the dynamic maintenance of these tumor states. During long-term evolution, tumor cells simultaneously activate several transcription factors associated with stemness and differentiation phenotypes, including SERPINH1 and ATF5^13–15^. Tumor cells with a high natural evolution signature, after injection into the brains of mice, have been found to regenerate a variety of cell states in the tissues, thereby increasing heterogeneity.

The high plasticity conferred by tumor evolution indicates that tumor cells can adapt to TME changes. Poor blood flow in the central necrotic areas of tumors creates a hypoxic environment. Our study^12^ has demonstrated that tumor cells activate the HIF1A/FOSL2 (AP-1 transcription factor subunit) regulatory axis under hypoxic conditions, thereby switching tumor states and remodeling the immunosuppressive microenvironment to support tumor development. GBM has also been found to induce WNT5A to mediate tumor cell differentiation into endothelium-like cells *via* the PAX6/DLX5 transcription program, and subsequently recruit peripheral endothelial cells, which form pseudo-blood vessels, thereby facilitating tumor-invasive growth^16^.

In response to treatment pressure, such as radiotherapy, tumor cells in the subventricular area have been found to activate an internal signaling pathway by binding CXCL12 in the microenvironment, thereby promoting the transformation of tumor cells into a mesenchymal-like state and facilitating their survival^17^. Such a state transition has recently been further confirmed by Wang et al.^18^, whose analysis of 86 primary-recurrent patient-paired GBM specimens with single-cell sequencing data has demonstrated that relapsed GBM is characterized by a shift to a mesenchymal-like state, which is mediated by AP-1. In addition, we have recently found that PTPRZ1-MET fusion and a high-frequency of MET exon 14 skipping (METex14) occur in brain metastases of lung cancer^19^. We also found that METex14 is present in a higher proportion (14%) of secondary glioblastomas than pan-gliomas (0.4%)^2^, thus suggesting that tumors bearing METex14 tend to occur in the brain microenvironment in advanced tumors. Consequently, these findings imply co-evolution of the tumor and microenvironment.

## Tumor evolution reconstructs the TME interaction network

**Figure 1:**
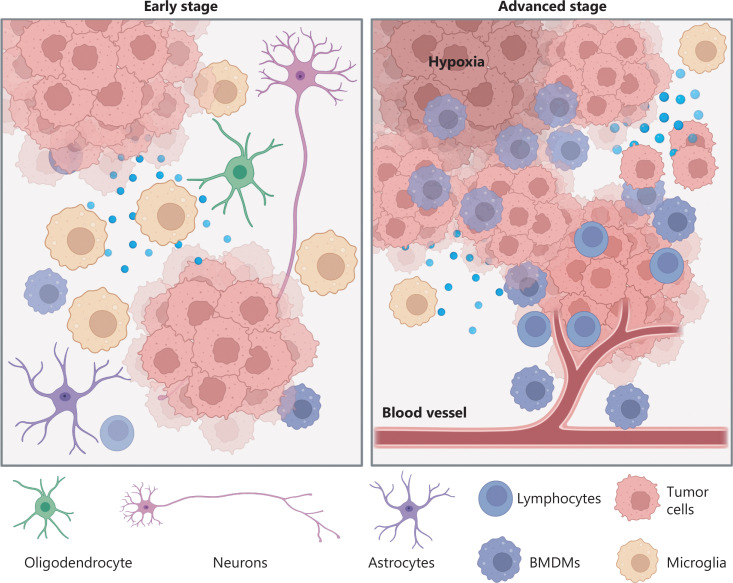
Coevolution of tumors and the tumor microenvironment. In early stages, tumor cells are often surrounded by neurons and glial cells. Crosstalk between tumors and the tumor microenvironment endows tumor cells with proliferation and invasion capabilities. In advanced stages, increased numbers of peripheral cells (e.g., BMDMs, lymphocytes, etc.) invade the tumor tissue. The high infiltration of BMDMs produces an immunosuppressive environment that further promotes tumor progression. Furthermore, hypoxic stress also accelerates tumor evolution.

The interaction between tumors and the TME evolves in tandem with tumor evolution (**Figure 1**). In a healthy brain, glial cells (oligodendrocytes, microglia, and astrocytes) perform various functions, such as axon myelination, immune surveillance, and blood-brain barrier (BBB) maintenance^20^. In the early stages of GBM evolution, microglia make up most macrophages and have proinflammatory roles through expressing cytokines such as TNF-α and IL-1β^21^, thus implying that they might play a role in activating inflammation and fighting tumor cells. However, evidence suggests that the IL-1β released by microglia binds the IL-1 receptor (IL-1R) on the surfaces of tumor cells and activates downstream pathways that promote tumor cell proliferation^22^. Yeo et al.^21^ have speculated that GBM stimulates the production and differentiation of brain resident oligodendrocytes. These oligodendrocytes in turn promote the invasiveness of GBM cells *via* the angiopoietin-2 signaling pathway^23^. Astrocytes also facilitate tumor cell infiltration by activating zinc finger E-box-binding homeobox 1 (ZEB1)^24^. Furthermore, astrocytes secrete proteases (uPA) that increase the expression of matrix metalloproteinases released by GBM cells, thus leading to extracellular matrix remodeling and promoting invasion^25^. Moreover, Venkataramani et al.^26^ have revealed that neurons have a typical synaptic ultrastructure located on tumor microtubes that generate postsynaptic currents mediated by AMPA subtype glutamate receptors on tumor cells, thereby promoting tumor growth and invasion.

The BBB, formed by the interplay of astrocyte foot processes with endothelial cells and pericytes, tightly regulates the inflow and outflow of substances to maintain a homeostatic environment for proper brain functioning^27,28^. However, tumor evolution may impair the BBB *via* inflammation, physical distortion, or increased vascularity, thus contributing to blood vessel leakage^29^. Consequently, many peripheral blood cells invade tumor tissue. Recent studies^12,21^ have confirmed that high infiltration of bone-marrow-derived macrophages (BMDMs) is associated with advanced evolution of GBM. Tumor cells may educate these BMDMs with multiple factors (e.g., CSF1), thus eventually forming an immunosuppressive microenvironment that inhibits T cell proliferation and interferon-gamma production. Other immune cells, such as regulatory T cells (Tregs), are also observed in high numbers in advanced stages of GBM progression^30^. Tregs are recruited to tumor tissue by CCL2/CCL22 and subsequently promote GBM progression. Tregs also inhibit the activity of T cells, and exert antitumor effects by secreting the cytokines IL-10 and TGF-β^31^. Furthermore, Zhang et al.^32^ have demonstrated that pericytes derived from the peripheral blood interact with tumor cells regardless of vascular structure. These pericytes activate the DNA damage repair pathway by secreting CCL5, which binds CCR5 on tumor cells, thus rendering glioma cells resistant to temozolomide.

## Current GBM evolutionary research strategies

Numerous studies have been conducted to deconvolve tumor evolution by investigating the characteristics of GBM (**Table 1**). Primary and recurrent GBM longitudinal cohorts are the classical model widely used to examine for tumor evolution driven by treatment^4,6,18,33–35^. For instance, Wang et al.^4^ have assessed the longitudinal genome and transcriptomic data of 114 patients, and have found that GBM has a highly branched evolutionary pattern during its recurrence: 63% of the patients experienced changes in GBM molecular subtypes. These studies have substantially deepened understanding of the tumor progression patterns driven by various treatments and have provided essential guidelines for the clinical management of recurrent GBM.

**Table 1:** Strategies for exploring tumor evolution

Strategy	Highlight	Reference
Longitudinal cohorts	A model widely used for identifying the differences between primary and recurrent GBMs, to explore treatment-driven tumor evolution	Kim et al.^3^ Wang et al.^4^ Kim et al.^6^ Wang et al.^18^ Johnson et al.^33^ Osuka & Van Meir^34^ Varn et al.^35^
Computational methods based on massive data	Computational models for predicting potential evolutionary sequences on the basis of large amounts of data	Ozawa et al.^36^ Korber et al.^37^
Multifocal GBMs	A model for identifying differences between lesions in the same GBM, to explore natural tumor evolution	Wu et al.^12^ Abou-El-Ardat et al.^38^ Liu et al.^39^ Lee et al.^40^
Organoids	A rapid model for exploring cellular crosstalk during tumor evolution; potentially limited by the complexity of cell populations and the tumor microenvironment	Tang et al.^41^ Zhang et al.^42^ Jacob et al.^43^
Animal models	Models for studying tumor evolution in animals	Yeo et al.^21^ Tentler et al.^44^ Miyai et al.^45^

Ozawa et al.^36^ have predicted the variation order in GBM through data modeling of massive GBM genomic variation, thus uncovering features in the natural evolution of primary GBM. Compared with other genomic alterations, chromosome 7 amplification and chromosome 10 deletion occur early in tumor evolution. Korber et al.^37^ have used a similar strategy to confirm this finding. Furthermore, their analysis has revealed that mutation of the TERT promoter is a prerequisite for rapid tumor growth. Although these computational methods provide powerful tools for dissecting driver events in tumor evolution, focusing on tumor genomic traits may fail to interrogate the dynamic changes in the microenvironment.

Multiple lesions from the same clonal ancestor can develop in a time- and space-independent manner, thus providing snapshots that reflect the different stages of tumor evolution^12,38–40^. Abou-El-Ardat et al.^38^ have found that, in advanced stages of GBM, aberrations in the RTK/PI3K, p53, and RB regulatory pathways are common and may be the factors driving subcloning. Our recent study has further examined multifocal GBMs with single-cell RNA sequencing and identified a positive feedback interaction between tumor cells and macrophages during GBM evolution^12^. Tumor cells in advanced stages of evolution recruit and polarize macrophages through ANXA1. These recruited BMDMs then secrete cytokines that promote tumor invasion and evolution.

Other strategies have leveraged the advantages of organoids^41–43^ or animal models^21,44,45^. Tang et al.^41^ have developed a rapid three-dimensional bioprinting method to integrate tumor cells and other stroma and immune cells for co-culture; this method may serve as a platform for studying cellular crosstalk during tumor evolution. Yeo et al.^21^ have used a mouse GBM model and single-cell RNA sequencing to characterize the multifaceted roles of macrophages from early to late stages of GBM evolution.

## Future perspectives

Owing to the inherent differences in the microenvironment among tumor regions, the heterogeneity of tumor cells among these regions is particularly pronounced. Puchalski et al.^46^ have constructed an anatomical transcription map of GBM and have found that mesenchymal-like tumor cells are enriched mainly in hypoxic and necrotic areas. In contrast, classical-like tumor cells are associated with vascular proliferation and infiltration areas. Thus, this preference for tumor cell enrichment could eventually result in a distinct crosstalk pattern between tumors and the tumor microenvironment across tumor regions. Future research should incorporate spatial factors into the interactions to thoroughly investigate cell communication, particularly that mediated by paracrine and direct signaling.

Beyond cell-to-cell communication, metabolism has been found to be an important factor in regulating interaction networks^47^. Altered metabolism in the tumor microenvironment can lead to the production of extracellular metabolites that alter the states and phenotypes of various cells. For instance, the GBM-derived metabolite KYN is involved in switching TAMs from the M1 to the M2 phenotype by activating the transcription factor AHR^48^. Glycolysis has been found to alter the microenvironment by producing lactic acid, which regulates T cell immune activity^49^. Consequently, dissecting the components of metabolites in the microenvironment will be crucial for establishing the potential links between tumors and the tumor microenvironment.

Given the spatial heterogeneity and dynamic changes in the tumor microenvironment alongside tumor evolution, the interplay between tumors and the tumor microenvironment is complex and regulated by multiple factors, including stresses, etc. Advances in single-cell technologies, such as single-cell tracking technology^50^ and single-cell spatial omics^51,52^, have led to potential clues for in-depth analysis of tumor-TME coevolution, and may contribute to better understanding of the mechanisms underlying cancer evolution.
